# Downstream Purification Strategies for Virus-like Particles: A Systematic Review of Structure Preservation, Impurity Control, and Viral Safety

**DOI:** 10.3390/microorganisms14040858

**Published:** 2026-04-10

**Authors:** Jingchao Zhang, Chen Chen

**Affiliations:** 1 College of Ecology and Environment, Chengdu University of Technology, Chengdu 610059, China; 2 School of Chemical Engineering and Technology, Tianjin University, Tianjin 300072, China; 15222599851@163.com

**Keywords:** VLPs, downstream purification, chromatography, process analytical technology, virus clearance, critical quality attributes

## Abstract

Virus-like particles (VLPs), nanoscale self-assembled structures lacking viral genetic material, have emerged as a versatile platform for vaccines, targeted delivery systems, and gene-editing applications owing to their strong immunogenicity, favorable biosafety profile, and high engineerability. However, the complex architecture of VLPs, their significant size heterogeneity, and the diversity of process- and product-related impurities generated in different expression systems make downstream purification a major bottleneck limiting product quality, yield, and manufacturability. This review systematically discusses advanced downstream purification strategies for VLPs from the perspective of three major objectives: preservation of structure and biological activity, control of product heterogeneity, and assurance of viral safety. First, strategies for maintaining VLP integrity and function are examined, including optimization of solution conditions, adoption of gentle yet efficient separation operations, and integration of process analytical technology (PAT) to reduce process-induced damage. Second, the review summarizes multi-step purification approaches—spanning clarification, ultrafiltration/diafiltration (UF/DF), chromatography, and disassembly/reassembly—to remove host cell proteins, host cell DNA, and product-related impurities while improving particle homogeneity and stability. Third, viral safety is discussed primarily from the perspective of downstream virus clearance under host-dependent risk, with particular attention to orthogonal clearance steps tailored to VLP properties and expression systems such as CHO cells and insect cell–baculovirus platforms. Overall, this review provides a CQA-oriented framework and practical guidance for the development of robust, scalable, and GMP-compliant downstream purification processes for VLP-based products.

## 1. Introduction

Virus-like particles (VLPs) are nanoscale multimeric structures self-assembled from viral structural proteins in heterologous expression systems. They closely mimic native virus particles in morphology and antigenicity but lack the viral genome, rendering them non-infectious and non-replicative [1,2]. VLPs can be enveloped or non-enveloped, with proteins arranged in single or multiple layers. Enveloped VLPs typically consist of a matrix protein core surrounded by a host-derived lipid membrane embedded with glycoproteins [3]. Historically, hepatitis B virus surface antigen particles were the first discovered VLPs [4], and over 100 different VLPs have now been produced and characterized [5].

The core advantages of VLPs lie in their native conformational antigen presentation and self-adjuvant effects. Their repetitive epitope structures efficiently activate B cells, inducing high-titer neutralizing antibodies, while also enabling cross-presentation via MHC I molecules to activate CD8^+^ T cells [6]. This makes them not only suitable as vaccines for preventing various infectious diseases [7,8], such as human papillomavirus (HPV), HBV, influenza, and SARS-CoV-2 but also expands their use into therapeutic vaccine areas, such as targeting key pathological molecules like β-amyloid (Aβ) in Alzheimer’s disease, the angiotensin system related to hypertension, and tumor-associated antigens (TAAs) [9,10,11]. Furthermore, leveraging their hollow interior and modifiable surfaces, VLPs can encapsulate drugs, nucleic acids, or proteins, and incorporate targeting ligands for tissue-specific delivery [12,13,14]. Compared to synthetic nanocarriers, VLPs inherit natural viral entry mechanisms while remaining fully biodegradable [15]. Recently, VLPs have also emerged in gene editing, enabling transient delivery of mRNA and RNP with reduced off-target effects [16,17,18,19,20,21,22]. With the continuous development of the biopharmaceutical industry, scientists are increasingly considering VLP-based formats during early-stage drug design or delivery system selection.

VLPs can be produced in a wide range of expression systems, including bacteria, yeast, insect cells, mammalian cells, plants, and cell-free platforms [23]. *E. coli* is often selected for proteins that do not require complex post-translational modifications (PTMs) [24]. Yeast is widely regarded as a cost-effective production host and has already enabled licensed VLP vaccines such as Engerix-B and Gardasil [25]. However, the limited PTM repertoire of microbial hosts constrains their suitability for some structurally complex or enveloped VLPs [26]. In contrast, mammalian cells and the insect baculovirus expression vector system (IBEVS) support more complex folding and PTMs, making them particularly useful for demanding non-enveloped and enveloped VLPs [27,28,29]. Cell-free and in vitro assembly platforms have also emerged as powerful alternatives, especially for rationally designed protein nanostructures and modular VLP-like systems [30,31,32,33]. Because most VLPs used in research and industrial development are generated by recombinant DNA expression in heterologous hosts, upstream construct design and downstream purification are closely interconnected. The expression construct, host platform, and secretion behavior can all influence particle assembly, impurity composition, and the suitability of subsequent purification strategies.

From an industrial perspective, VLP platform selection and downstream process design must also consider cost-effectiveness, since low productivity, complex impurity profiles, and the need for multiple purification steps can markedly increase manufacturing costs [34,35]. In practice, downstream processing remains one of the major bottlenecks limiting the productivity, quality consistency, and manufacturability of VLP-based products. Compared with conventional soluble recombinant proteins, VLPs are larger, structurally more complex, and often more heterogeneous. Their process streams may contain high levels of host cell proteins (HCPs), host cell DNA (HCD), extracellular vesicles, viruses, partially assembled intermediates, aggregates, or encapsulated impurities, depending on the production route [36,37,38]. Moreover, VLPs can be highly sensitive to pH, ionic strength, shear stress, interfacial exposure, and concentration-dependent instability during purification [39,40]. Importantly, these challenges are not uniform across VLP products, but vary substantially with the production host, assembly mode, and recovery route.

A further complication is that VLP downstream processing does not follow a single manufacturing paradigm. Instead, the nature of the feed stream and the logic of purification depend strongly on the expression host, intracellular or extracellular assembly behavior, and the intended process strategy. One important distinction relates to feed origin: intracellularly produced non-enveloped VLPs generally require cell disruption and therefore generate lysates rich in nucleic acids, cell debris, membrane fragments, and misassembled species [41]. In contrast, secreted or budded VLPs from mammalian or insect systems are typically recovered from culture supernatants, where the impurity burden is often dominated by soluble host-derived components, extracellular vesicles, and, in IBEVS processes, baculovirus [42]. A second distinction relates to purification logic. Some processes aim to preserve intact VLPs throughout downstream processing, whereas others intentionally disassemble VLPs into smaller subunits to remove internal impurities before controlled reassembly [41,43]. These route-dependent paradigms differ fundamentally in feed properties, major process risks, suitable unit operations, and analytical requirements.

**Figure 1:**
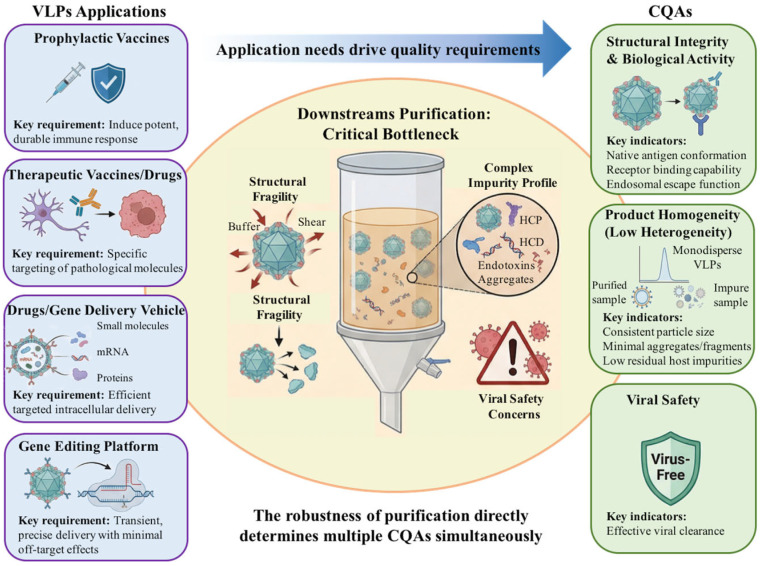
Quality-by-Application strategy for downstream purification of VLPs. The specific requirements of VLPs in four major application areas—prophylactic vaccines, therapeutic drugs, delivery vehicles, and gene-editing platforms—define three critical quality attributes (CQAs): structural integrity and biological activity, product homogeneity, and viral safety. Downstream purification is crucial for ensuring these CQAs but faces challenges such as structural fragility and complex impurity profiles.

**Figure 2:**
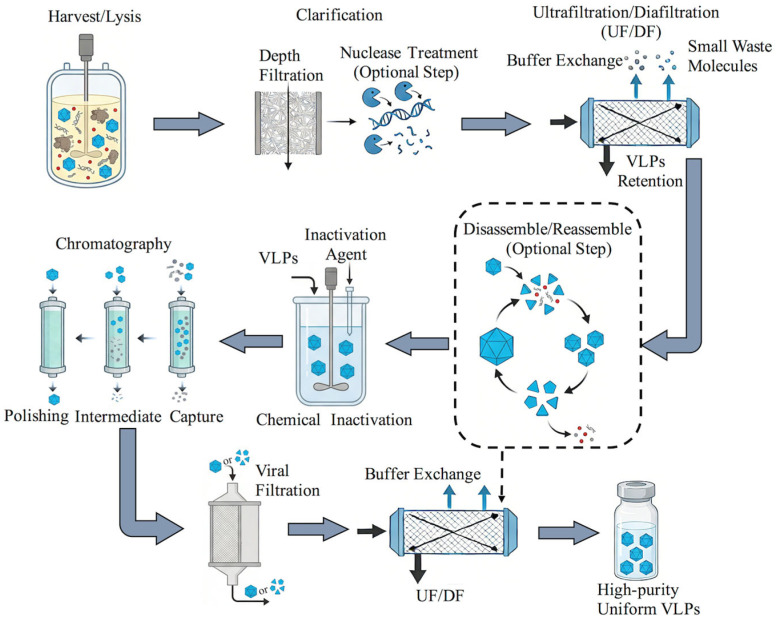
Modular process framework for downstream purification of VLPs. Depending on the production route, VLP downstream processing may begin either from clarified culture supernatant or from cell lysis followed by clarification. Subsequent operations can include nuclease treatment, ultrafiltration/diafiltration (UF/DF), chromatography-based capture and polishing, optional disassembly/reassembly for selected non-enveloped VLPs, chemical inactivation where appropriate, virus filtration, and final UF/DF for formulation. The figure represents a route-dependent framework rather than a fixed universal sequence.

For these reasons, VLP downstream development must be understood as a quality-driven and route-dependent engineering problem. The key critical quality attributes (CQAs) of VLPs generally include: (1) preservation of structural integrity and biological activity, (2) control of product heterogeneity, and (3) assurance of viral safety when required by the production system. These quality attributes directly affect efficacy, safety, stability, delivery performance, and regulatory acceptability. To illustrate this quality-oriented perspective, a conceptual framework for VLP downstream purification is summarized in Figure 1. In addition, to provide a process-level guide for the discussion that follows, an integrated but modular overview of VLP downstream purification strategies is presented in Figure 2. This workflow is not intended to represent a single universal sequence; rather, it highlights how actual process design may differ depending on feed type, particle fragility, impurity profile, and whether purification is based on intact-particle preservation or disassembly/reassembly logic.

Against this background, rather than simply cataloging established unit operations, this review integrates them into a VLP-specific downstream framework centered on critical quality attributes (CQAs) and route-dependent process contexts. Specifically, the review is organized around three industrially decisive objectives: preservation of structural integrity and biological activity, control of product heterogeneity, and assurance of viral safety. It further examines how these objectives are shaped by different production paradigms, including intracellular versus secreted feed streams, intact-particle purification versus disassembly/reassembly routes, and host-specific differences for viral safety such as CHO-based mammalian expression and insect-cell-based IBEVS. The review also highlights the enabling role of process analytical technology (PAT) in translating these quality-oriented concepts into robust, scalable, and GMP-aligned manufacturing strategies. In this way, it aims to provide both a conceptual framework and a practical reference for the rational design of VLP downstream purification processes.

## 2. Purification Strategies for Maintaining the Structure and Activity of VLPs

The biological performance of VLPs depends fundamentally on the preservation of their higher-order structure. Their immunogenicity, receptor recognition, delivery behavior, and formulation stability are all closely linked to particle integrity, surface organization, and assembly state. However, as supramolecular complexes composed of multiple protein subunits—and in some cases lipid envelopes—VLPs are intrinsically more vulnerable than many soluble recombinant proteins to environmental and process-induced stress. Changes in pH, ionic strength, temperature, shear exposure, adsorption to interfaces, concentration polarization, or inappropriate residence in restrictive chromatographic pores may induce aggregation, partial disassembly, conformational deformation, or loss of function.

Before discussing individual downstream tools, it is important to distinguish two major process logics in VLP purification. The first is direct purification of intact VLPs, which is especially relevant for fragile enveloped particles and secretion-based processes, where preservation of native morphology is the primary objective from harvest onward. The second is disassembly-assisted purification, mainly applicable to selected non-enveloped VLPs, where transient conversion into smaller subunits enables removal of internalized nucleic acids or misassembled species, followed by controlled reassembly. These two routes impose different constraints on buffer design, chromatography selection, and process monitoring. Against this background, this section discusses three enabling strategies for protecting VLP structure and activity during downstream processing: rational buffer system design, chromatography media and transport regimes that reduce structural stress, and process analytical technology (PAT) for real-time quality surveillance.

### 2.1. Buffer System Optimization

**Figure 3:**
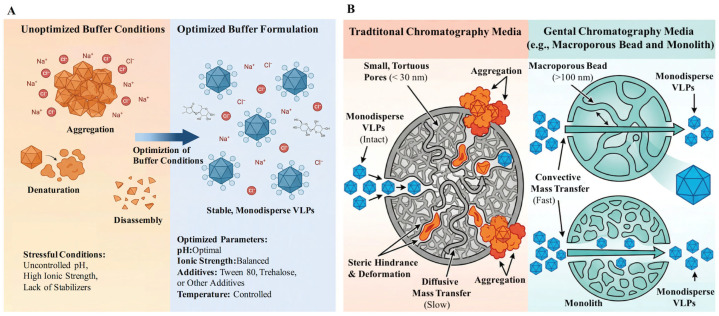
Strategies for maintaining structure and activity during VLP purification. (**A**) Buffer condition optimization: a comparison shows that unoptimized harsh conditions (uncontrolled pH/ionic strength, no stabilizers) lead to VLP aggregation, denaturation, or disassembly. In contrast, optimized conditions involving controlled pH, balanced ionic strength, addition of stabilizers (e.g., Tween 80, trehalose), and temperature control yield stable, monodisperse VLPs. (**B**) Comparison of chromatographic media mass transfer mechanisms: traditional small-pore media (pore size < 30 nm) cause VLP entrapment, aggregation, and deformation, relying on slow diffusive mass transfer. In contrast, gentle media such as macroporous beads and monoliths (pore size > 100 nm) allow VLPs to remain monodisperse, enabling rapid convective mass transfer which minimizes shear stress and structural damage.

Buffer system design for VLP purification should be guided by general physicochemical principles rather than empirical case-by-case optimization alone. Because VLPs are multimeric supramolecular assemblies, their stability depends on the balance among electrostatic repulsion, hydrophobic interaction, subunit-interface strength, and, for enveloped VLPs, membrane integrity [44]. Accordingly, buffer development should systematically evaluate: (1) pH, which affects both capsid protein ionization and conformational stability; (2) ionic strength and ion species, which can either suppress nonspecific interactions or promote salt-induced aggregation; (3) excipients such as nonionic surfactants that reduce interfacial stress during pumping, filtration, or storage; and (4) stabilizers such as sugars or polyols that improve thermal and freeze–thaw robustness (Figure 3A) [41,45,46,47,48]. A useful starting point is to identify the dominant failure modes of the target particle under likely process stresses, including pH and conductivity shifts, temperature changes, repeated freeze–thaw exposure, mechanical agitation, and concentration steps. Orthogonal analytical tools such as dynamic light scattering (DLS), electron microscopy, HPLC, and antigenicity assays can then be used to detect aggregation, degradation, conformational drift, or loss of assembly [49,50,51]. This stress-informed characterization is particularly valuable because the stability of VLPs cannot usually be inferred from a single condition alone and may depend on both particle type and solution context. Buffer optimization should therefore prioritize the identification of key variables while also recognizing that pH, salt type, conductivity, and additive selection may need to be assessed as an interrelated design space during later-stage process development.

Representative studies illustrate this principle. For bacteriophage MS2 VLPs, a 100 mM NaNO_3_–Tris buffer at pH 8.0 improved performance as a delivery platform by suppressing aggregation and maintaining a uniform particle size of 27–30 nm for at least 12 months at 4 °C, whereas Tris or HEPES alone led to poorer stability [51]. Norovirus VLPs also show genotype-dependent responses to ionic environment: GII.17 particles remained stable across a range of ionic strengths, while GII.4 aggregated under low ionic strength conditions [52]. For enveloped systems, the margin of stability may be even narrower. HIV-1 envelope VLPs formed aggregates larger than 300 nm in PBS after one week at room temperature, illustrating how seemingly benign formulation conditions may still be incompatible with long-term particle stability [53]. These examples also highlight why buffer optimization must extend across the full downstream process rather than being treated only as a formulation task. VLPs are exposed to multiple unit operations, hold steps, and concentration changes, and both intermediates and final products may evolve even during short storage intervals [54]. Once preliminary buffer conditions have been identified, they should therefore be validated under realistic process scenarios, including concentration stress, thermal acceleration, and repeated freeze–thaw cycles. For example, physiological ionic strength can induce aggregation of HPV VLPs, whereas optimization to 0.22 M NaCl combined with 0.01% Tween 80 maintained particle monodispersity [55]. The likely mechanism is mitigation of interfacial adsorption and nonspecific particle–particle interactions, thereby preserving particle homogeneity. Similarly, a lyophilization protectant consisting of 7.5% trehalose together with Tween 80 and glycine significantly improved the stability of foot-and-mouth disease virus (FMDV) VLPs over the temperature range from 4 °C to 37 °C [56].

The design implications are route-dependent. In intact-particle purification, especially for enveloped VLPs, buffer conditions should remain as conservative as possible in order to preserve both capsid architecture and membrane organization from harvest onward. By contrast, in disassembly/reassembly-based routes, controlled shifts in pH, ionic strength, or redox environment may be intentionally introduced to trigger reversible structural transitions. In such cases, the objective is not to avoid all perturbation, but to define a reversible operating window that supports disassembly for impurity removal while preserving the capacity for efficient reassembly and restoration of function. Thus, buffer development in VLP downstream processing is not merely supportive formulation work, but a central engineering tool that links particle physicochemical behavior to process robustness.

### 2.2. Gentle Chromatography Techniques

Traditional methods like precipitation and gradient density centrifugation, while usable at lab scale, suffer from low recovery, limited impurity removal, and poor scalability. Meanwhile, such methods are cumbersome, time-consuming, and not conducive to process scaling or large-scale production [57,58]. In recent years, chromatography has become a primary development direction for VLP purification. Studies show that rational application of chromatography can fully leverage process potential, obtaining VLPs with higher quality and better activity [59]. However, commonly used agarose-based media have pore sizes typically <30 nm, close to the size of many VLPs. This pore size limitation prevents large molecules from entering the media interior, leading to low binding capacity [60]. Furthermore, narrow pores force VLPs to rely primarily on slow diffusive mass transfer, reducing throughput and potentially causing structural deformation upon adsorption [61]. Therefore, selecting gentle chromatography strategies is not only an important means for process scale-up but also a key pathway to maintain VLP structural integrity and ensure their biological activity and immunogenicity (Figure 3B).

Macroporous chromatography media with pore sizes > 100 nm or even 200 nm address these limitations. Compared to traditional small-pore media, they not only significantly improve static and dynamic binding capacity, accelerate mass transfer rates, and increase recovery but, more importantly, are gentler on VLP structure, effectively maintaining particle integrity, reducing protein unfolding, and better preserving biological activity. In the purification of foot-and-mouth disease VLPs (diameter ~28 nm), using DEAE-FF media with an average pore size of 32 nm made it difficult for virus particles to enter pores and prone to clogging and degradation into immunogenically weaker 12S pentamers, reducing active recovery. Switching to DEAE-650M and DEAE-POROS media with pore sizes of 106 nm and 214 nm, respectively, allowed intact virus particles to pass through smoothly, reduced diffusion resistance, and significantly improved particle structure, thermal stability, and recovery, with the largest-pore DEAE-POROS showing the best recovery [62]. Similarly, macroporous anion exchange media with average pore sizes ranging from 120 to 280 nm, such as DEAE-AP-280 nm, enabled HBV VLPs to fully diffuse into the inner pores, increasing the static adsorption capacity by 12.9-fold and the dynamic capacity by 8-fold. The stability of VLPs was significantly improved, with 85.5% of VLPs correctly self-assembling at a load of 2 mg/mL. This difference was clearly observable via confocal laser scanning microscopy [61]. Recently, the cellulose-based “Monolith-Like Particles” (MLP) developed by the Kadoi team further extended this concept. Their through-pore radius reached 1.5 μm, and after modification with dextran sulfate, they were used for influenza virus and SARS-CoV-2 purification [63,64]. These findings support a general design principle: for intact VLP capture, pore accessibility is often a prerequisite for both productivity and structural preservation. However, as larger or more fragile enveloped VLPs emerge, developing new-generation macroporous media remains a key challenge.

Membrane chromatography combines the selectivity of chromatography with high-throughput membrane separation, using functionalized macroporous membranes (0.65–3 μm pores) as the matrix. Due to open pore channels, mass transfer is primarily convective: small impurities penetrate deep into pores while larger VLPs rapidly complete adsorption at pore surfaces. This mode offers high throughput and speed, while significantly reducing time VLPs are confined in narrow pores or exposed to shear stress [65]. For structure-sensitive particles, such convective architectures can reduce both processing time and hydrodynamic stress. For example, the HIV-1 structural protein Gag can assemble in vitro into enveloped VLPs approximately 100–150 nm in diameter, which are extremely sensitive to shear stress. Studies show that using anion exchange membranes for their concentration and purification not only effectively recovers the target product but also, as confirmed by cryo-transmission electron microscopy, preserves their intact enveloped spherical structure [29,66]. Similarly, in adenovirus purification with a similar structure, a metal affinity membrane chromatography system constructed by immobilizing Zn^2+^ ions on a membrane matrix showed significantly higher average yield than traditional resin chromatography and exhibited excellent ability to distinguish intact viruses from defective particles, achieving efficient polishing [67]. These results illustrate why membrane chromatography is often particularly attractive for large enveloped VLPs and other particles that are difficult to handle using bead-based systems.

Monolithic columns consist of a single piece of highly interconnected porous network with micrometer-scale channels, offering high porosity, low back pressure, and suitability for high-throughput operation. These characteristics are particularly beneficial for the rapid, gentle purification of large molecular particles like viruses, VLPs, and even more fragile exosomes [68,69,70,71]. For example, in the purification of HBV VLPs, using a hydroxyl-derived monolithic column based on hydrophobic interaction chromatography achieved a dynamic binding capacity three times higher than traditional bead resins, with a recovery of 90%. Transmission electron microscopy and atomic force microscopy characterization confirmed that purified VLPs maintained correct structure and particle size [72]. For more shear-sensitive enveloped VLPs, the gentle fluid environment of monolithic columns is even more applicable. For instance, in the purification of SARS-CoV-2 VLPs expressed using the insect baculovirus system, a combination of hydrophobic and cation-exchange monolithic columns for capture and polishing steps yielded VLPs that fully reproduced the natural virus’s structural and morphological features. This purified product, combined with an adjuvant, successfully induced strong neutralizing antibody responses in mice, highlighting the key role of monolithic columns in preserving VLP immunogenicity [73].

Furthermore, other emerging novel media in recent years offer more possibilities for VLP purification. For example, non-woven structures composed of cellulose nanofibers prepared by electrospinning feature open sub-micron fiber networks and high specific surface area. Their primarily convective mass transfer mechanism allows high flow rate operation, greatly shortening processing time, and has been validated in adeno-associated virus (AAV) downstream purification [74]. Another type, medium–large pore hydrogel microspheres based on poly (ethylene glycol) diacrylate (PEGDA), formed via low temperature photopolymerization with an average pore size of about 400 nm, can achieve rapid, gentle capture of fragile vesicles like exosomes [75]. Although these systems remain less mature in VLP manufacturing, they illustrate the same underlying trend: future chromatographic platforms will increasingly be judged by how well they combine selectivity with accessibility, convective transport, and low structural stress.

Overall, the chromatographic strategy for VLP purification should be framed as a balance between separation performance and particle preservation. In direct purification of intact particles—particularly for enveloped VLPs or large non-enveloped assemblies—large-pore and convective formats are often preferred because they reduce pore restriction, shorten residence time, and lower the risk of structural damage. In disassembly/reassembly-based processes, where the target may temporarily exist as smaller subunits, a broader range of media may become feasible, and chromatographic selection can be more strongly driven by impurity removal objectives. The information on selected commercially available macroporous chromatographic media is summarized in Table 1.

### 2.3. Process Analytical Technology (PAT)

In VLP downstream purification, real-time and accurate process analysis is crucial for maintaining particle structural integrity and biological activity. Traditional offline analytical methods, including Electron Microscopy, DLS, HPLC, SDS-PAGE, and ELISA, can assess final product particle size, purity, and activity, but their “sample-analyze-wait” mode suffers from significant lag. Once quality anomalies are detected, the batch has often deviated irreversibly, causing substantial time and economic losses [76]. Therefore, process analytical technology (PAT) emerged. This systematic framework was formally proposed by the US FDA in 2002 within its initiative “Pharmaceutical cGMPs for the 21st Century-A Risk-Based Approach.” It aims to “build quality in” throughout the product manufacturing process by monitoring and controlling critical process parameters (CPPs) and critical quality attributes (CQAs) in real time, rather than relying solely on final product testing [77]. PAT has become a key tool in biopharmaceuticals. By integrating analytical equipment online or at-line into the production process, it enables real-time, continuous, non-destructive monitoring of CQAs, significantly enhancing process control and data reliability [78]. In the context of VLP downstream processing, its importance lies in enabling the timely detection of route-specific failure modes, including aggregation during concentration, structural perturbation during capture or polishing, incomplete disassembly or reassembly, and the emergence of altered particle populations that may not be evident from bulk concentration measurements alone.

Integration of spectroscopic technologies is an important PAT means, favored for their non-invasiveness, rapid response, and multi-component simultaneous detection capabilities [79]. For example, a Raman-based PAT platform has been successfully used to monitor the precipitation process of HBV VLPs, enhancing robustness against process fluctuations and effectively preventing VLP co-precipitation or irreversible aggregation [80]. NIR spectroscopy is often integrated into UF/DF steps, using calibration models to monitor concentration changes in feed and retentate in real time, warning of precipitation or aggregation risks due to high concentration [81]. UV/VIS spectroscopy is suitable for real-time tracking of concentration dynamics in target product and impurity conjugate systems, providing immediate feedback for process control [82,83].

Online chromatography provides high-resolution solutions for monitoring purity, charge, size variants, glycosylation, and other complex attributes. Through automated sampling systems, samples can be automatically delivered to chromatographic detection modules. Studies have coupled online HPLC with rapid size-exclusion chromatography (SEC) to simultaneously monitor high molecular weight (HMW) aggregates and critical excipient concentrations during monoclonal antibody UF/DF steps, with accuracy comparable to offline methods [84]. This strategy can be directly transferred to VLP buffer exchange processes for real-time assessment of exchange efficiency and aggregation tendency. Furthermore, HPLC systems designed for process analysis can complete sampling and analysis in 1–3 min, suitable for monitoring column dynamic capacity and product variants [85].

Physical and particle characterization techniques provide direct insights into VLP assembly state. Dynamic light scattering (DLS) analyzes fluctuations in scattered light intensity over time, enabling online monitoring of hydrodynamic diameter and distribution, particularly suitable for tracking assembly/disassembly kinetics. Static light scattering (SLS) measures angular dependence of scattered light to obtain molecular weight information, qualitatively reflecting assembly progress. For example, during the disassembly and reassembly of HBV VLPs, combined DLS and SLS techniques enabled continuous online monitoring of assembly rate and disassembly extent, filling a gap in process analysis for this step [86,87]. Multi-angle light scattering (MALS) directly determines molecular weight and aggregation state and is a key tool for monitoring HMW aggregates, playing an important role in polishing steps [88].

In recent years, emerging biosensors, such as those based on surface plasmon resonance or affinity recognition, can provide highly specific detection of particular CQAs like HCPs and HCD, though their robustness in large-scale production still requires verification [76]. PAT, by integrating spectroscopy, chromatography, and light scattering, enables dynamic monitoring of CQAs during VLP purification. This allows timely correction of process deviations, ensures batch-to-batch consistency, and provides indispensable support for maintaining VLP structural integrity and biological activity. With advancements in automation and data integration, PAT will play an increasingly central role in scaled-up VLP production.

## 3. VLP Heterogeneity Management

Preservation of VLP structure and biological activity, as discussed above, is only one prerequisite for product quality. A second major downstream challenge is product heterogeneity, which directly influences safety, efficacy, stability, and regulatory acceptability. In VLP manufacturing, impurities can be broadly divided into two categories. Process-related impurities include host cell proteins (HCPs), host cell DNA (HCD), medium components, detergents, and column leachables [70,89,90], some of which may contribute to immunogenicity or other safety concerns [91,92,93]. Regulatory agencies accept risk-based control approaches for these impurities [94]. Product-related impurities, by contrast, originate from the target itself and include unassembled subunits, aggregates, and degradation fragments [95,96,97]. These species are particularly challenging because they may share substantial physicochemical similarity with intact VLPs while lacking the intended structural and immunological properties.

For this reason, VLP downstream purification must be designed not only to remove host- and process-derived contaminants, but also to resolve particle-related variants that reflect incomplete or aberrant assembly. In practice, this requires integration of multiple operations, including clarification, ultrafiltration/diafiltration (UF/DF), chromatography, and, where appropriate, disassembly/reassembly-based purification. The relative importance of these operations depends strongly on the production route. Intracellular platforms generate lysate-derived impurity burdens that differ substantially from those of secretion-based systems, while disassembly-assisted purification introduces additional opportunities for removal of internalized impurities and misassembled species. Against this background, the following section discusses how major downstream unit operations contribute to heterogeneity control in route-dependent VLP purification processes.

### 3.1. Clarification

Clarification is the first major step in impurity burden reduction and serves to remove cell debris, large particulates, and other insoluble material before higher-resolution downstream operations. Although often viewed as a preparative step, clarification has important implications for both impurity control and process robustness, because the quality of the clarified feed directly affects filter fouling, membrane performance, and chromatographic selectivity in subsequent steps. The design of clarification is strongly influenced by the cellular localization of the product. For intracellularly expressed VLPs, clarification must be preceded by cell disruption to release the target product [98]. The lysis strategy should be selected according to host type and product fragility, since it shapes both the impurity profile and the risk of particle damage. Chemical lysis using detergents is often applied in mammalian or insect systems and may be acceptable for certain non-enveloped VLPs [99], whereas mechanical methods such as high-pressure homogenization or sonication are more commonly used for microbial hosts including bacteria and yeast [100]. In the industrial production of HPV and HBV VLPs in yeast, for example, high-pressure homogenization is widely used for product release [101,102]. In all such cases, protease inhibitors may be required to limit degradation during and after lysis [103].

**Figure 4:**
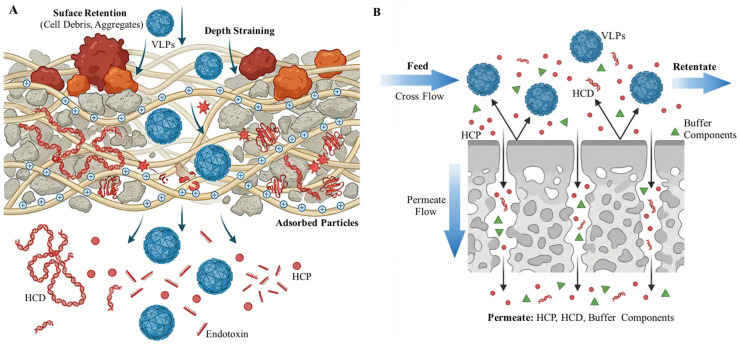
Strategies for depth filtration and UF/DF separation mechanisms during VLP purification. (**A**) Deep filtration mechanism: Through a combined action of surface retention, depth sieving, and charge adsorption, it effectively removes impurities such as cell debris, HCD, HCP, and endotoxins while preserving the target VLPs. (**B**) UF/DF mechanism: The feed flows parallel to the membrane surface. Small-molecule impurities (HCP, HCD, buffer components) pass through as permeate, while VLPs are retained and concentrated in the retentate, achieving both concentration and buffer exchange.

Current mainstream clarification technologies primarily include centrifugation and depth filtration. Batch centrifugation is common at the lab scale but offers limited removal of fine impurities due to density-dependent separation. In contrast, depth filtration has become the preferred option for large-scale production. It is not mere sieving but utilizes a porous filter bed to achieve deep retention of fine particles and some soluble impurities through a combination of size exclusion, electrostatic adsorption, and hydrophobic interactions, significantly reducing impurity load on downstream chromatography columns (Figure 4A) [104]. Depth filter media mainly consist of cellulose or polypropylene fibers, embedded with filter aids like diatomaceous earth or perlite to provide a vast adsorption surface area. These materials are bound by charged polymer resins, enabling filters to specifically capture oppositely charged impurities via electrostatic interactions—e.g., positively charged resins adsorb negatively charged HCPs, HCD, and endotoxins [105]. Research data confirms their efficacy. For instance, a commercial anion exchange depth filter achieved 3 log_10_ removal of DNA and 7 log_10_ removal of endotoxin [106]. In another case, a fully synthetic depth filter exhibited high binding capacity for HCPs [107]. Additionally, depth filters are often used as prefilters before virus removal filtration to enhance overall process robustness [108,109].

Following clarification, nuclease treatment degrades residual host nucleic acids into harmless fragments, reducing immunogenicity risks and eliminating nucleic acid entanglement that could impair chromatographic resolution. Practice shows that optimized nuclease treatment can degrade over 90% of residual DNA and significantly improve the downstream purification of products like HIV VLPs [110,111]. From the perspective of heterogeneity management, clarification and nuclease treatment together help create a cleaner and more uniform feed stream, thereby reducing the risk that downstream steps must resolve complex mixtures of VLPs, nucleoprotein complexes, and cell-derived particulate contaminants. In secretion-based routes, clarification is generally milder and primarily serves to remove cells, debris, and extracellular particulates from culture supernatant; in intracellular routes, by contrast, it plays a much larger role in managing the extensive impurity burden generated by lysis.

### 3.2. UF/DF

UF/DF is a key unit operation in VLP purification, using membranes with specific pore sizes to achieve concentration, buffer exchange, and impurity removal based on physical sieving (Figure 4B) [112]. Its practical role, however, depends strongly on the production route. In secretion-based processes, UF/DF often serves as an early concentration step to reduce feed volume and remove impurities before chromatography [89]. In intracellular or lysate-derived processes, it may play a larger role in removing nucleic acid fragments, detergents, or salts after clarification or disassembly [37]. In disassembly/reassembly workflows, UF/DF can become a central transition tool for exchanging from disassembly-promoting conditions into reassembly-promoting buffers [43].

Ultrafiltration membranes typically have an asymmetric structure, with their separation function primarily relying on a dense surface skin layer with precise pore size selectivity. Common membrane materials include polyethersulfone (PES), regenerated cellulose, and polyvinylidene fluoride (PVDF) [113], primarily in flat-sheet cassette and hollow fiber formats [114]. By leveraging molecular weight differences between VLPs and impurities, UF/DF selectively removes small-molecule impurities. Since correctly assembled VLPs are very large, using a membrane with an appropriate molecular weight cutoff (e.g., 100–1000 kDa) can efficiently retain target particles while allowing smaller impurities like HCPs, HCD fragments, residual detergents, lipids, and product degradation fragments to pass through [115,116]. Experimental data fully confirms its effectiveness. For instance, in influenza VLP purification, using a 1000 kDa ultrafiltration membrane removed 62.3% of HCPs and 33.3% of HCD [117]. In an AAV purification case, a 500 kDa ultrafiltration membrane effectively removed 98% of HCPs [118]. Furthermore, by optimizing solution conditions, such as salt concentration, during UF/DF, the removal of HCPs and other impurities can be enhanced, providing cleaner loading samples for downstream polishing chromatography steps [119]. UF/DF also enables process-economical advantages, for example, by pre-concentrating feed before nuclease treatment and thereby reducing the consumption of expensive reagents [120]. At the final stage, it is commonly used to exchange purified VLPs into the formulation buffer required for storage and administration [121]. However, fluid shear forces generated during UF/DF can pose challenges to VLP structural integrity and biological activity. Improper operating parameters may lead to particle aggregation or structural damage [70]. Therefore, process optimization is crucial. This involves the systematic screening of core parameters such as membrane material, format (particularly hollow fiber formats, which offer lower shear stress), molecular weight cutoff (MWCO), operating pressure, and shear rate. Studies show that the activity of enveloped HIV Gag-VLPs can be well maintained under optimized low-shear conditions [122]. Conversely, higher shear stress can lead to significant aggregation during room temperature storage [123].

### 3.3. Chromatography

While clarification and UF/DF serve important roles in process integration and initial concentration, they have limited capacity for deeply removing key impurities like HCPs, HCD, aggregates, and unassembled monomers. High-resolution chromatography is therefore essential, offering excellent separation efficiency under gentle conditions that maintain VLP biological activity [59]. Chromatography separates based on differences in physicochemical properties between target molecules and impurities, including size, charge, hydrophobicity, and biospecific affinity [124]. In VLP purification, typically, two or three different chromatography modes are combined to form capture, intermediate purification, and polishing steps.

**Figure 5:**
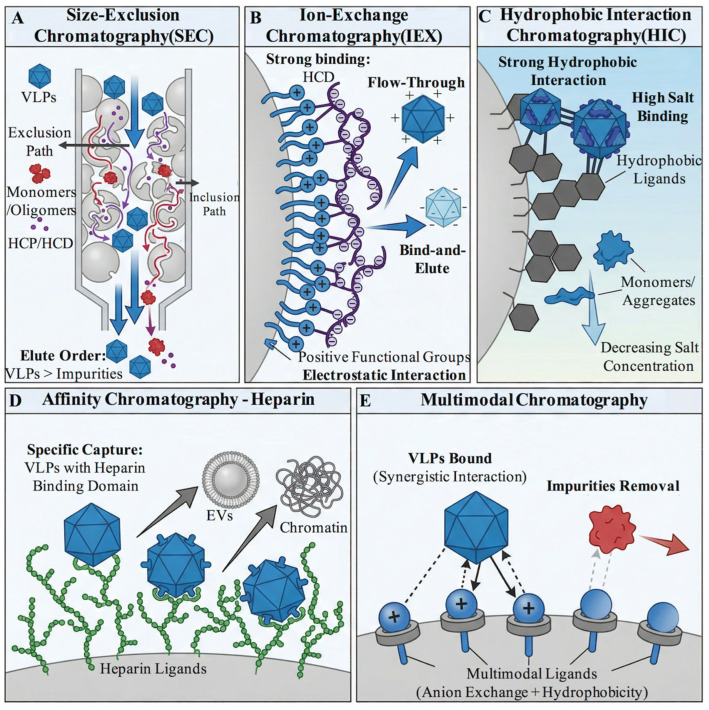
Schematic illustration of separation principles for five chromatography techniques used in VLP purification. (**A**) Size-exclusion chromatography (SEC): Separation is based on hydrodynamic volume differences. Intact VLPs, being too large to enter the pores, elute first through the void volume. Smaller impurities such as unassembled monomers, oligomers, HCP, and HCD enter the pores and are delayed. (**B**) Ion-exchange chromatography (IEX): Separation relies on differences in surface charge. In the flow-through mode of anion exchange chromatography (AEX), strongly negatively charged impurities (e.g., HCD) are tightly adsorbed by positively charged media, while target VLPs flow through due to charge differences. In bind-elute mode, VLPs with different charges can be selectively eluted by adjusting salt concentration. (**C**) Hydrophobic interaction chromatography (HIC): Separation depends on differences in surface hydrophobicity. VLPs bind to the media via hydrophobic interactions under high salt conditions and are eluted upon decreasing salt concentration. Unassembled monomers or aggregates exhibit different elution behaviors due to varying hydrophobicity. (**D**) Heparin Affinity Chromatography: This technique captures VLPs via specific interaction between heparin ligands and heparin-binding domains on certain VLP surfaces. Impurities lacking this domain (e.g., extracellular vesicles, chromatin) do not bind, enabling highly selective purification. (**E**) Multimodal Chromatography: The media ligands integrate multiple interaction modes (e.g., ion exchange and hydrophobicity). This synergistic effect enhances the binding selectivity for VLPs and simultaneously allows for effective removal of various “stubborn” impurities with similar physicochemical properties.

Size-exclusion chromatography (SEC) separates analytes on the basis of hydrodynamic size and is often useful as a polishing tool for removing low-molecular-weight impurities and resolving assembled VLPs from unassembled proteins or smaller oligomers (Figure 5A). Because intact VLPs are much larger than most soluble proteins, SEC can provide clear discrimination between assembled particles and monomeric or oligomeric capsid components. In HBV VLP purification, appropriately selected SEC media effectively separated fully assembled particles from unassembled capsid protein species, thereby improving overall purity [125]. SEC has also been used to remove HCPs and HCD during HIV VLP purification [89]. However, despite its separation clarity, SEC is constrained by low loading capacity, long processing times, and poor scalability, with practical loads typically limited to approximately 5% of column volume or less [126]. As a result, it is most often reserved for analytical use or final polishing rather than large-scale capture.

Ion-exchange chromatography (IEX) is among the most widely used chromatographic modes in VLP purification because it offers strong resolving power for both host-derived impurities and selected particle variants (Figure 5B). Separation is based on differences in net surface charge and can be operated in bind-elute or flow-through mode depending on the relationship between the target and impurity profiles. Anion exchange chromatography (AEX) is especially effective for removal of HCD, since nucleic acids are strongly negatively charged and usually bind more strongly than proteins under relevant process conditions. By adjusting pH and conductivity, it is often possible to allow VLPs either to flow through or to elute under relatively mild salt conditions while retaining residual HCD on the medium, thereby markedly reducing nucleic acid burden [127,128,129]. Beyond HCD clearance, IEX can also contribute to removal of HCPs, extracellular vesicles, incomplete particles, and aggregates [130,131,132,133,134,135]. For many processes, IEX therefore serves as a versatile bridge between bulk impurity reduction and higher-resolution polishing.

However, when certain impurities have charge properties extremely similar to the target, hydrophobic interaction chromatography (HIC) may be an effective solution (Figure 5C). HIC relies on interactions between hydrophobic regions on molecule surfaces and hydrophobic ligands on the stationary phase. This interaction strengthens at high ionic strength and weakens at low salt [136]. Studies show that intact VLPs, due to their highly ordered surface topology, exhibit stronger overall hydrophobicity than their constituent monomers. For example, in HBV VLP purification, researchers computationally and experimentally confirmed their surface as “strongly hydrophobic,” enabling intact particles to effectively bind to HIC media, while free epitope peptides or unassembled proteins were removed during loading or low-salt elution, yielding VLPs with nearly 100% purity [137]. Furthermore, multiple studies indicate that HIC also performs well in removing HCPs, HCD, and vesicle-like impurities [138,139,140].

Affinity chromatography provides the highest selectivity among common chromatographic modes by exploiting specific biological interactions such as antibody–antigen or receptor–ligand recognition. Its use in VLP purification is therefore especially attractive when highly similar host-derived particles or product-related variants are difficult to separate by conventional physicochemical methods. Heparin affinity chromatography is one prominent example. Because heparin can interact specifically with surface domains present on some viral particles, it can enable selective capture of target VLPs while allowing many unrelated impurities to pass through (Figure 5D) [141,142]. In HIV-1 Gag VLP purification, heparin affinity chromatography has been shown to separate target particles from host-derived extracellular vesicles and chromatin [66]. Related applications have also been reported for measles virus and chimeric influenza VLP purification [143,144]. However, affinity approaches are inherently target dependent, and heparin is useful only for VLPs that present compatible binding motifs [145]. In this context, recombinant engineering can expand the applicability of affinity chromatography by introducing or reshaping surface features that support selective capture. For example, inserting a surface-exposed His6 tag into the VP1 G-H loop enabled one-step immobilized metal affinity purification of foot-and-mouth disease virus, with marked reductions in host cell proteins and residual DNA [146]. Beyond metal-chelate systems, capsid engineering has also been used to confer new affinity properties in other vectors; an engineered AAV8 capsid was reported to acquire both heparin and AVB Sepharose binding capacity, illustrating that rational sequence modification can create affinity handles even when native ones are absent [147]. Nevertheless, such strategies remain product-dependent and must be balanced against possible effects on assembly, biological activity, and product quality. For this reason, affinity chromatography is best regarded as a high-value, product-specific option rather than a universal platform.

Multimodal chromatography has developed rapidly as a means of resolving impurities that are difficult to remove using any single interaction mechanism. These media combine two or more interaction types—such as ion exchange, hydrophobic interaction, hydrogen bonding, metal coordination, or thiophilic interaction—within a single ligand architecture (Figure 5E). Such combined selectivity can be particularly valuable in separating “stubborn” impurities or product-related variants that overlap strongly with the target in size or charge [136]. For example, multimodal cation-exchange media have shown strong performance in removing unassembled monomers and aggregates [127,148,149]. Core700 media operated in flow-through mode can significantly reduce HCPs, HCD, and small-molecule impurities [70], while multimodal anion exchangers and hydroxyapatite media have also been applied in the purification of several VLP systems [150,151]. As VLP products become more diverse and impurity profiles more complex, multimodal media are likely to play an increasing role in polishing steps that require simultaneous control of process impurities and structural heterogeneity.

### 3.4. Disassembly and Reassembly

During assembly, VLPs may encapsulate impurities like HCPs, HCD, or lipids within their interior [152,153,154]. Such internal impurities cannot be directly removed by conventional chromatography or UF/DF and may pose safety risks. To address this, disassembly and reassembly steps can be introduced into the downstream purification process: under specific conditions, VLPs are disassembled into monomeric proteins, releasing internal impurities. These impurities are then removed via chromatography or other purification steps, followed by buffer exchange via UF/DF to allow monomeric proteins to reassemble into VLPs. This approach is mainly applicable to selected non-enveloped VLPs whose assembly is reversible under controlled physicochemical conditions. For example, for HBc-MAGE3 I VLPs, an optimized disassembly/reassembly process combined with nuclease treatment successfully produced VLPs with >90% purity and free of nucleic acids [155]. *Acinetobacter* phage AP205 VLPs also achieved >90% RNA removal via a urea-based disassembly/reassembly step [153]. This process not only effectively clears internal impurities but can also improve VLP morphological homogeneity and stability. For instance, HBV VLP proteins expressed in *E. coli* often form high molecular weight species (HMWS). After disassembly/reassembly, HMWS content was effectively reduced, yielding more homogeneous VLPs [42]. Similarly, for triple-layered rotavirus VLPs, the outermost VP7 protein is prone to detachment, degrading triple-layer VLPs into double-layer VLPs and VP7 protein, affecting product yield and quality. By controlling ionic strength, temperature, and calcium ion conditions, disassembly and reassembly can be achieved, with glycerol added to stabilize the triple-layer structure [156].

Therefore, developing efficient disassembly and reassembly processes is important. A successful example is HPV VLPs using this step to enhance homogeneity, stability, and immunogenicity: first, under low ionic strength and high pH (e.g., pH 8.2), low concentrations of reducing agents (e.g., DTT) are added, primarily disrupting disulfide bonds maintaining VLP structure. The reassembly process involves removing the reducing agent using methods such as dialysis or UF/DF, lowering pH (to 6.0–7.0), and increasing ionic strength (e.g., 0.5–1 M NaCl) to trigger spontaneous reassembly of pentameric subunits [157]. Numerous studies confirm that many VLPs can achieve efficient disassembly/reassembly by modulating physicochemical parameters. Success depends on precise control of disassembly driving forces and reassembly triggering mechanisms. Examples include MS2 bacteriophage VLPs [158], polyomavirus SV40 [159], parvovirus B19 (B19V) [160], hepatitis C virus (HCV) [161], and cowpea chlorotic mottle virus (CCMV) VLPs [162]. However, this strategy also presents challenges. Under critical conditions for disassembly or reassembly, protein subunits may undergo nonspecific aggregation due to exposed hydrophobic regions. For example, HBV VLPs at urea concentrations exceeding 4 M form disordered aggregates and cannot effectively reassemble [155].

## 4. Downstream Strategies for Assuring Viral Safety of VLPs

Biological products must be highly purified and free from microbial contamination, yet production using engineered cells or biological materials carries risks of adventitious virus contamination. Historical incidents—poliovirus vaccine contaminated with SV40 and plasma-derived products transmitting HIV—underscore the critical importance of viral safety [163,164]. Regulatory bodies including ICH, FDA, and EMA have established systematic viral safety standards based on three pillars: selecting low-risk starting materials, viral testing throughout production, and validating virus removal/inactivation steps in downstream processing [165,166,167,168,169]. Although this section focuses primarily on downstream virus removal and inactivation, the first two pillars remain essential components of the overall viral safety strategy for VLP products. In practice, careful control of cell substrates, raw materials, and ancillary reagents helps minimize the introduction of adventitious agents at source, while in-process and end-of-production viral testing provides evidence that the manufacturing system remains under control. These measures define the baseline viral risk that downstream clearance steps must ultimately address.

Compared with more mature biologics such as antibodies [170], viral clearance studies for VLPs are still insufficient. Differences in expression systems and VLP characteristics themselves add complexity to process design. Therefore, tailoring downstream virus clearance strategies for VLPs is particularly important. This section discusses downstream viral clearance considerations for different expression systems and explores strategy formulation based on VLP properties to enhance product safety. Note that specific methods and regulatory requirements for viral clearance process validation are relatively mature; relevant content should refer directly to current guidelines.

### 4.1. Downstream Viral Clearance Requirements for Different Expression Systems

Current main expression systems for VLPs include *E. coli*, yeast, CHO cells, and IBEVS. Since animal viruses cannot replicate in *E. coli* and yeast, downstream viral clearance processes are not required for these two systems [171,172].

CHO cells are the preferred host for industrial production of monoclonal antibodies (mAbs), Fc-fusion proteins, recombinant clotting factors, and novel vaccine antigens such as HIV envelope trimers and VLPs [28,173]. However, CHO cells carry the risk of expressing endogenous retrovirus-like particles (RVLPs) and may introduce adventitious viruses, for instance the minute virus of mice (MVM), via raw materials like serum or other additives [174,175]. Therefore, ensuring viral safety is a core requirement for CHO platform process development.

IBEVS has become an important platform for producing VLPs, subunit vaccines, and gene therapy vectors. It has been successfully used for commercial production of HPV VLPs, recombinant COVID-19 VLPs, and recombinant influenza vaccines [176,177]. However, IBEVS inevitably co-produces large quantities of infectious budded baculovirus (BV) during VLP production [178]. Additionally, researchers have found that the insect Sf9 cell line harbors a novel rhabdovirus (sf-rhabdovirus) [179], and the Hi5 cell line is commonly infected with alphanodavirus (TnCLV) [180]. Although no literature currently reports these viruses posing harm to human health, their potential risks still raise regulatory concerns about product safety. Recently, the ICH Q5A (R2) guideline was revised, explicitly stating: if baculovirus is used as a helper virus to infect cells for expressing the target protein, the purification process must include viral clearance steps and related validation [166].

### 4.2. VLP Downstream Viral Clearance Strategies

Regulations recommend incorporating at least two different viral clearance steps, one effectively clearing non-enveloped viruses, with complementary modes of action. Current downstream viral clearance processes primarily employ chemical methods, chromatography, and virus filtration [166].

Chemical inactivation uses chemical treatment to inactivate viruses. For recombinant protein products, common chemical methods include solvent/detergent (S/D) treatment and low pH incubation [181]. The S/D method typically uses detergents like Triton X-100 or Tween 80 at concentrations of 0.5–1.0%, combined with the organic solvent tri-n-butyl phosphate (TNBP) at 0.1–1% [175]. However, environmental concerns regarding Triton X-100 degradation products are driving searches for alternatives [182]. Low pH incubation is another viral inactivation method. Studies show that incubation at pH 3.5–3.8 for at least 60 min can effectively inactivate enveloped retroviruses, typically achieving >5 log_10_ reduction [183], primarily by inducing conformational changes in viral surface proteins [184]. Note that chemical methods mainly inactivate enveloped viruses, such as xenotropic murine leukemia virus (X-MuLV) and baculovirus [185,186], and have limited effect on non-enveloped viruses [187]. Furthermore, chemical methods often involve harsh conditions, such as extreme pH or the use of organic solvents, which may adversely affect VLP structure and biological activity while inactivating viruses. Therefore, before introducing such methods, their applicability must be systematically assessed by comparing the key quality attributes—structure, activity, and purity—before and after inactivation to determine the most suitable inactivation process and conditions.

Chromatography achieves virus removal based on physicochemical property differences between viruses and target products. Ion-exchange and multimodal chromatography are most widely used in VLP processes. Anion exchange chromatography plays a significant role in virus removal, especially in flow-through mode. The principle is that virus surfaces often carry strong negative charges and can be adsorbed by positively charged ligand media under low conductivity, while the target product flows through due to charge differences, achieving efficient separation [170]. Research data shows that this mode provides robust clearance for viruses like X-MuLV and MVM, typically achieving >4 log_10_ reduction [188]. For example, in HCV VLP purification, AEX in flow-through mode achieved 99% baculovirus clearance with 66% target product recovery [130]. Additionally, in bind-elute mode, if the isoelectric points of the virus and VLPs differ, effective separation can also be achieved by optimizing elution conditions. For instance, in influenza VLP purification, this mode achieved 4.3 log_10_ baculovirus clearance while recovering the target product [131]. Conversely, if the isoelectric points are similar, co-elution may occur, hindering virus removal [187]. Cation-exchange chromatography also shows excellent virus clearance capability under specific conditions. For example, in cGMP production of HIV nanoparticle immunogens, systematic optimization of chromatography conditions achieved >13 log_10_ clearance of enveloped viruses and >7 log_10_ clearance of non-enveloped viruses [189], further demonstrating the potential of ion-exchange chromatography for viral safety control. However, traditional IEX performance can be limited in high-salt or complex sample matrices. To overcome this, multimodal chromatography is a good option. For example, the multimodal anion exchange media Capto^TM^ adhere can effectively adsorb X-MuLV even at high NaCl concentrations, with better tolerance than traditional AEX media [190]. Similarly, the multimodal membrane chromatography Natrix^®^ CH, by introducing hydrophobic interactions, can operate stably at relatively high salt concentrations, particularly suitable for virus clearance in later VLP purification stages or high-salt systems [191].

Virus filtration removes viruses based on size exclusion using nanoscale porous membranes: target molecules smaller than the pores pass through while larger viruses are retained [192]. This gentle process efficiently clears both enveloped and non-enveloped viruses without damaging biologic activity. Information on some commercially available virus filters is shown in Table 2. Virus filters are mainly categorized into two types: large-pore filters with pores around 35 and 50 nm, primarily retaining larger retroviruses; and small-pore filters with pores around 15 and 20 nm, effectively removing a broader spectrum of viruses, including small non-enveloped viruses [109]. In mAb purification, filters with average pore sizes around 20 nm can achieve over 4 log_10_ reduction of the challenging small non-enveloped virus MVM [193].

However, for large molecular complexes like VLPs, their size may approach or exceed filter pore sizes. Therefore, targeted filtration strategies must be designed based on their specific characteristics, mainly falling into three scenarios: (1) VLPs can be reversibly disassembled: Typically, VLP size exceeds 20 nm, making direct passage through 20 nm virus filters difficult. If VLPs can be disassembled into smaller monomeric proteins under specific conditions, they can pass through 15–20 nm small-pore filters and later be reassembled into intact particles. This is the most ideal scenario, as small-pore filters offer excellent removal for both large and small viruses, a capability well exemplified in the HPV VLP purification process [194]. (2) VLPs are 20–50 nm in size and cannot be disassembled: In this case, 35 or 50 nm large-pore filters must be used, allowing VLPs to pass while retaining larger viruses. AAV purification is a typical application of this strategy [195]. However, such filters have limited capability against small viruses, requiring complementary clearance via chromatography steps. (3) VLPs are too large and cannot be disassembled: When VLP size far exceeds the retention range of commercial filters, conventional virus removal filtration may not be directly applicable. In this situation, communication with regulatory authorities is needed to discuss product-specific considerations and evaluate comprehensive strategies. These may include using specific expression systems not requiring downstream viral clearance, such as *E. coli* or yeast, or modifying VLP properties through molecular design to meet filtration process requirements, such as by reducing VLP size or by enabling “triggerable disassembly” [196,197,198]. All strategies must prioritize ensuring product safety, efficacy, and other CQAs.

## 5. Conclusions and Perspectives

The broad application potential and structural complexity of VLPs impose unusually high demands on downstream purification. This review systematically examined downstream process design from the perspective of three interrelated objectives: preservation of structure and biological activity, control of heterogeneity, and assurance of viral safety. Across these dimensions, a central conclusion is that successful purification of VLPs cannot rely on any single unit operation, but instead requires integrated, product-specific process design guided by the critical quality attributes of the target particle. Maintaining native VLP structure and function depends on the careful control of solution conditions, use of gentle yet effective separation methods, and increasing incorporation of PAT for real-time monitoring and mitigation of process-induced damage. Achieving high purity and particle homogeneity requires coordinated multi-step strategies, in which clarification, UF/DF, and especially chromatography play central roles in removing host cell proteins, host cell DNA, and product-related impurities. For selected VLPs, disassembly/reassembly offers a distinctive route not only for improving homogeneity and stability, but also for removing internal or tightly associated impurities, although its applicability remains product-dependent and is currently most mature for certain non-enveloped systems. Ensuring viral safety adds another layer of complexity, particularly for products derived from mammalian or insect expression systems. As emphasized in this review, the practical focus for VLP purification is often the design of orthogonal downstream virus clearance steps that are compatible with product CQAs. Chromatography frequently provides a broadly compatible removal mechanism, whereas chemical inactivation and virus filtration require more careful case-by-case evaluation because the same physicochemical features that define VLP function may also constrain viral clearance feasibility. At the same time, these downstream measures should be understood within a broader viral safety framework that also includes low-risk starting materials and appropriate viral testing throughout manufacturing.

Future developments in VLP downstream purification will likely follow these trends: (1) Continuous innovation in media and materials, such as smart responsive materials and media with precisely controlled properties driven by 3D printing technology [199,200]. Furthermore, artificial intelligence (AI) and machine learning (ML) are being used to predict structure–performance relationships of porous materials, accelerating new material screening [201]. (2) Process intelligence and continuous manufacturing, deeply integrating quality by design (QbD) principles [202], PAT, and automated control to transition from batch to continuous manufacturing, improving production efficiency and process robustness. (3) Parallel development in regulatory science, establishing clearer standards for identifying product- and process-related impurities and guidelines for viral safety assessment tailored to VLP characteristics, supporting efficient development and review of these innovative drugs. In summary, deep understanding of product characteristics and rational application of integrated strategies are essential to overcome purification bottlenecks and enable high-quality, safe, and effective VLP-based products.

## Figures and Tables

**Table 1:** Several commercial macroporous chromatography media.

Name	Pore Size	Principle	Type	Manufacturer
POROS™ 50HQ	50~100 nm	Anion exchange	Resin	Thermo Fisher
Toyopearl^®^ NH(2)-750F	≥100 nm	Anion exchange	Resin	TOSOH
Macro-Prep^®^ High Q	100 nm	Anion exchange	Resin	Bio-Rad
POROS™ 50HS	50~100 nm	Cation exchange	Resin	Thermo Fisher
Toyopearl^®^ SP-650C	100 nm	Cation exchange	Resin	TOSOH
Macro-Prep^®^ High S	100 nm	Cation exchange	Resin	Bio-Rad
Toyopearl^®^ Phenyl FT-750F	≥100 nm	Hydrophobic interaction	Resin	TOSOH
CHT™ Type I	60~80 nm	Multimodal	Resin	Bio-Rad
CHT™ Type II	80~100 nm	Multimodal	Resin	Bio-Rad
POROS™ Heparin	50~100 nm	Affinity	Resin	Thermo Fisher
Mustang^®^ Q	0.8 μm	Anion exchange	Membrane	PALL
Sartobind^®^ Q	3~5 μm	Anion exchange	Membrane	Sartorius
Natrix^®^ Q	0.4 μm	Anion exchange	Membrane	Merck Millipore
3M™ Polisher ST	0.8 μm	Anion exchange	Membrane	3M
Mustang^®^ S	0.65 μm	Cation exchange	Membrane	PALL
Natrix^®^ CH	1.0 μm	Cation exchange	Membrane	Merck Millipore
Sartobind^®^ S	3~5 μm	Cation exchange	Membrane	Sartorius
Sartobind^®^ Phenyl	3~5 μm	Hydrophobic interaction	Membrane	Sartorius
CIMmultus^®^ QA	2.0 μm	Anion exchange	Monolith	Sartorius
CIMmultus^®^ DEAE	2.0 μm	Anion exchange	Monolith	Sartorius
CIMmultus^®^ EV	2.0 μm	Anion exchange	Monolith	Sartorius
CIMmultus^®^ SO3	2.0 μm	Cation exchange	Monolith	Sartorius
CIMmultus^®^ OH	2.0 μm	Hydrophobic interaction	Monolith	Sartorius
CIMmultus^®^ PrimaS	2.0 μm	Multimodal	Monolith	Sartorius

**Table 2:** Several commercial virus filters.

Manufacturer	Name	Pore Size	Format	Material	Target Virus
PALL	Pegasus™ SV4	20 nm	Flat sheet	PVDF	Small virus
PALL	Pegasus™ Prime	20 nm	Flat sheet	PES	Small virus
PALL	Ultipor™ VF Grade DV50	50 nm	Flat sheet	PVDF	Large virus
PALL	Ultipor™ VF grade DV20	20 nm	Flat sheet	PVDF	Small virus
Merck Millipore	Viresolve^®^ Pro	N/A	Flat sheet	PES	Small virus
Merck Millipore	Viresolve^®^ NFR	78 nm	Flat sheet	PES	Large virus
Sartorius	Virosart^®^ HF	20 nm	Hollow fiber	PES	Small virus
Sartorius	Virosart^®^ CPV	20 nm	Flat sheet	PES	Small virus
Asahi Kasei	Planova™ 15N	15 nm	Hollow fiber	CRC	Small virus
Asahi Kasei	Planova™ 20N	19 nm	Hollow fiber	CRC	Small virus
Asahi Kasei	Planova™ 35N	35 nm	Hollow fiber	CRC	Large virus
Asahi Kasei	Planova™ BioEX	N/A	Hollow fiber	PVDF	Small virus

N/A: not available. Abbreviations: PES, polyethersulfone; PVDF, polyvinylidene chloride; CRC, cuprammonium-regenerated cellulose.

## Data Availability

No new data were created or analyzed in this study. Data sharing is not applicable to this article.
